# Interaction effects of sedentary behavior and depression on MAFLD in NHANES 2017–2020 and 2021–2023

**DOI:** 10.1371/journal.pone.0342336

**Published:** 2026-02-17

**Authors:** Wuqian Zhang, Yujing Wang, Qunyou Cong, Xiaoshun Tang, Zhe Wang, Zhirong Wang

**Affiliations:** 1 Department of Gastroenterology, Shanghai Pudong Hospital, Fudan University Pudong Medical Centre, Shanghai, China; 2 School of Medicine, Tongji University, Shanghai, China; 3 Department of Gastroenterology, Tongji Hospital, Tongji University, Shanghai, China; Chung Shan Medical University, TAIWAN

## Abstract

**Background:**

Metabolic-associated fatty liver disease (MAFLD), a disease closely associated with metabolic abnormalities (e.g., insulin resistance, dyslipidemia, elevated blood glucose, and obesity), has become a global public health challenge. This study explored the independent associations between sedentary behavior and depressive symptoms with MAFLD and analyzed the potential interactions between these two factors and the risk of MAFLD occurrence.

**Methods:**

This cross-sectional study is based on data from two cycles of the National Health and Nutrition Examination Survey (NHANES) from 2017 ~ 2020 and 2021 ~ 2023 and analyzes the relationships among depressive symptoms, sedentary behavior, potential confounding factors, and MAFLD. A multivariate logistic regression model was used in the present study, and different subgroups of sedentary behavior were analyzed to assess the associations among sedentary behavior, depression and MAFLD. In addition, the presence of additive interactions among these variables was assessed by calculating the relative excess risk due to interaction (RERI), attributable proportion due to interaction (AP), and synergy index (S).

**Results:**

A total of 10,612 participants were included in this study (weighted sample size of 183,231,593), of whom 4,501 (weighted proportion of 42%) had MAFLD. Compared with participants with less daily sedentary time, those with more sedentary time had a greater risk of MAFLD (OR = 1.33, 95% CI: 1.08 ~ 1.64). We also found that, compared with individuals without depressive symptoms, individuals with depressive symptoms were significantly associated with an increased risk of MAFLD (OR = 1.26, 95% CI: 1.03 ~ 1.54). Furthermore, sedentary behavior significantly interacted with depression in overweight individuals, affecting the risk of MAFLD (relative excess risk (RERI) = 0.684, 95% CI: 0.312 ~ 1.057; attributable proportion (AP) = 0.360, 95% CI: 0.323 ~ 0.397; synergy index (S) = 1.623, 95% CI: 1.177 ~ 2.070). Poisson regression with 90% CI confirmed this interaction (RERI = 0.318, 90% CI: 0.036 ~ 0.600; AP = 0.223, 90% CI: 0.182 ~ 0.263; S=1.310, 90% CI: 1.000 ~ 1.620).

**Conclusion:**

Sedentary behavior and depression have a significant synergistic effect on MAFLD in overweight individuals. Therefore, improving individuals’ psychological health status and reducing sedentary time may have positive effects on the prevention and treatment of MAFLD.

## Background

Metabolic-associated fatty liver disease (MAFLD), formerly known as nonalcoholic fatty liver disease (NAFLD), is a chronic liver disease associated with metabolic disorders characterized by the pathological accumulation of triglycerides and other lipids in hepatocytes [1]. Currently, approximately one-third of the global population is affected by this disease, making it a major challenge in the field of global public health [2]. The disease can progress from simple hepatic steatosis to steatohepatitis and ultimately progress to end-stage liver disease [3]. MAFLD is closely related to systemic energy metabolism disorders, including insulin resistance and dyslipidemia [4].

Depression, also known as depressive disorder, is a common mood disorder characterized by persistent low mood, loss of interest or pleasure, thereby reducing quality of life [5]. In the United States, according to data from the National Health and Nutrition Examination Survey (NHANES), depression is independently associated with MAFLD and the degree of hepatic fibrosis after excluding clinically relevant confounding factors [6].

Sedentary behaviors refer to the postures of sitting, leaning, or lying while awake, with low energy expenditure, usually not exceeding 1.5 times the basal metabolic rate [7]. Sedentary behaviors include but are not limited to working while sitting for long periods, driving, watching TV, or other screen-based entertainment activities [8]. This lifestyle is becoming more common in modern society, and many people spend most of their day sitting due to changes in work patterns, the development of transportation, and the diversification of entertainment methods. Previous studies have shown that sedentary behavior is associated with the occurrence of various metabolic-related diseases [9,10].

Therefore, if MAFLD occurs simultaneously with depression or sedentary behavior, two different risk combinations may be formed, emphasizing the importance of identifying depression or sedentary behavior in MAFLD patients. A meta-analysis of sedentary behavior and depression revealed a significant positive correlation between sedentary behavior and the risk of depression (RR = 1.31, 95% CI: 1.16 ~ 1.48) [11]. Currently, relatively few studies have investigated the interactive effects of depression and sedentary behavior with MAFLD. Most previous studies have used only NHANES data from 2017 ~ 2018, whereas this study includes the latest released data from 2021 ~ 2023, thereby expanding the sample size. Therefore, this study aimed to explore the joint impact of depression and sedentary behavior on MAFLD through NHANES data.

## Methods

**Study Population:** This is a cross-sectional study with a complex, multistage probability sampling design based on data from the National Health and Nutrition Examination Survey (NHANES). The NHANES is a nationwide study that tracks the health and nutritional status of adults and children in the United States. The survey uses a complex, multistage probability sampling design to sample participants and oversamples minorities and the elderly to make the results applicable to other populations. To obtain a larger sample for analysis, we combined NHANES data from 2017 ~ 2020 and 2021 ~ 2023. The ethics review committee of the National Center for Health Statistics approved the ethics of the study. All individuals signed written informed consent before participating in the study. More information about the NHANES can be found on the NHANES official website: http://www.cdc.gov/nhanes [12].

**Fig 1:**
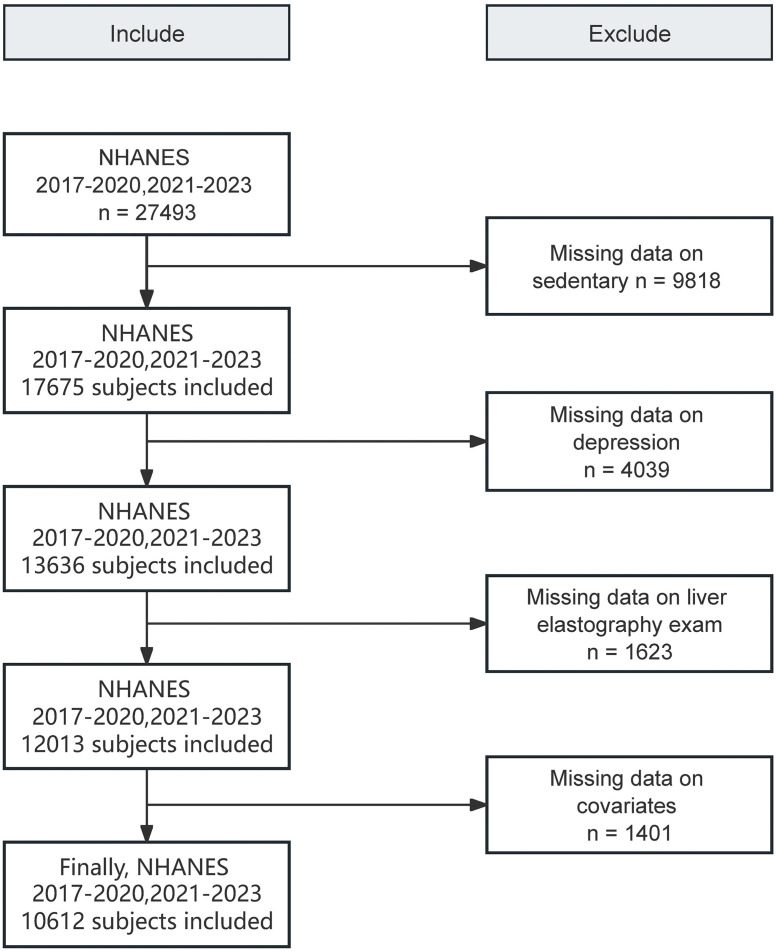
Flowchart of the selection process used in this study.

Out of 27,493 participants extracted from the NHANES database, we excluded those with missing data on sedentary behavior (n = 9,818), depression questionnaires (n = 4,093), liver elasticity examinations (n = 1,623), and other covariate information (n = 1,401). A total of 10,612 participants were included in this study. The workflow of this study is shown in Fig 1.

**Outcome Variable:** The outcome variable in this study was the presence of MAFLD in participants, which was categorized as a binary variable (presence/absence). The current diagnostic criteria for MAFLD positivity are based on evidence of hepatic steatosis, in addition to one of the following three criteria: overweight/obesity, presence of type 2 diabetes mellitus (T2DM), or evidence of metabolic dysregulation [13]. Metabolic dysregulation includes the following: (1) waist circumference > 102 cm for men or > 88 cm for women; (2) blood pressure > 130/85 mmHg or use of anti-hypertensive medication; (3) blood triglycerides > 150 mg/dL or use of lipid-lowering medication; (4) plasma high-density lipoprotein cholesterol (HDL-C) < 40 mg/dL for men and < 50 mg/dL for women; (5) prediabetes (fasting blood glucose 100 ~ 125 mg/dL or glycated hemoglobin 5.7 ~ 6.4%); (6) homeostatic model assessment of insulin resistance score ≥ 2.5 points; and (7) plasma high-sensitivity C-reactive protein (hsCRP) levels > 2 mg/L [13]. According to the European Association for the Study of the Liver (EASL), the controlled attenuation parameter (CAP) > 274 dB/m is used as the cutoff threshold for identifying hepatic steatosis in this study [14]. CAP is a non-invasive measurement index derived from transient elastography (a type of ultrasound-based examination), which quantifies the degree of hepatic steatosis by assessing the ultrasound attenuation of liver tissue. In simple terms, it reflects the “fat content” in the liver—higher CAP values indicate a greater accumulation of fat in liver cells.

**Explanatory variables:** The independent variables in this study are sedentary behavior and depression. Sedentary behavior is collected on the following question: “How much time do you usually spend sitting on a typical day?”. Sedentary behavior is defined as the total time spent sitting per day, in hours/day. Sedentary time < 2 hours/day is considered short sedentary time, 2 ~ 6 hours/day is considered moderate sedentary time, and > 6 hours/day is considered long sedentary time. Depressive symptoms are tested through the Patient Health Questionnaire (PHQ-9), which is a 9-item screening tool that asks about the frequency of depressive symptoms in the past two weeks. The total PHQ-9 score ranges from 0 to 27, with 0–4 points (no depression) as the reference group. Specifically, 0–4 points indicates no depression, 5–9 points indicates mild depression, and > 9 points indicates moderate or severe depression. A PHQ-9 score of ≥ 5 points is considered to indicate the presence of depression symptoms [15].

**Covariates:** The selection of covariates was based on variables identified in the existing literature as being associated with the outcome variable (MAFLD) [16–19]. The following categorical covariates were included in our analysis: sex, age, race, education level (≤ high school and >high school), smoking status (never, current and past smokers), body mass index (BMI, normal or lighter weight groups (BMI < 25 kg/m^2^), overweight groups (25 kg/m^2^ ≤ BMI < 30 kg/m^2^), obese groups (BMI > 30 kg/m^2^)), and diabetes. The following continuous covariates were included in our analysis: plasma high-density lipoprotein cholesterol (HDL-C), total cholesterol (TC), and high-sensitivity C-reactive protein (hsCRP). Sex, age, and race are self-reported demographic information. BMI was calculated by dividing the participant’s weight by the square of their height (kg/m^2^). Education level is assessed by the following question: “What is the highest grade or level of school you have completed? What is the highest degree you have received?”. Participants are identified as current or past smokers by asking if they have ever smoked 100 cigarettes in their lifetime and if they currently smoke. The participants were considered diabetic if they met any of the following criteria: (1) had a glycated hemoglobin concentration > 6.5% or a fasting blood glucose level > 126 mg/dL; (2) answered “yes” to the question “Has a doctor ever told you that you have diabetes?” or “Are you currently taking insulin?”

**Missing Data Handling:** After excluding 9,647 participants aged < 18 years, missing data in this study included sedentary behavior, depression (PHQ-9 score), liver elasticity, and some covariates (e.g., educational level, smoking status, BMI). Given that key indicators such as liver transient elastography were non-randomly missing (selective detection), the complete case analysis (CCA) might introduce potential bias by excluding over half the sample and violating the Missing Completely At Random (MCAR) assumption. To address this limitation and verify the robustness of CCA results, multiple imputation (MI) was performed as a sensitivity analysis using the MICE package in R software. The MI process was designed as follows: 1) All the core variables mentioned earlier were included in the imputation model; 2) Imputation methods were tailored to variable types: predictive mean matching (PMM) for continuous variables (e.g., PHQ-9 score, hsCRP), logistic regression for binary variables (e.g., MAFLD, diabetes), and polytomous logistic regression for categorical variables (e.g., depression severity, smoking status); 3) Five imputed datasets were generated, and each dataset was analyzed using the same weighted logistic regression model as CCA (accounting for NHANES complex sampling design: strata, clusters, and sampling weights); 4) Results from the five imputed datasets were pooled using Rubin’s rules to calculate combined effect sizes (odds ratios, ORs) and 95% confidence intervals (CIs).

**Data analysis:** The quantitative data are presented as the means and standard deviations (S.Ds.), and the weighted t test was used for comparisons between the MAFLD and non-MAFLD groups. Categorical variables are described by case numbers and weighted prevalence [n (weighted%)], and differences between the MAFLD and non-MAFLD groups were assessed via the chi-square test. Multivariate logistic regression analysis was used to test the relationships among sedentary behavior, depression, and MAFLD. Model 1 is the unadjusted model. Model 2 adjusts for demographic confounders (sex, age, race, and education level), as these variables are fundamental characteristics that may potentially influence both exposures and the outcome and are commonly adjusted for in MAFLD-related studies [16]. Model 3 is the fully adjusted model, which, in addition to the variables adjusted in Model 2, also adjusts for BMI, diabetes status, smoking status, and plasma high-density lipoprotein cholesterol (HDL-C), total cholesterol (TC), and high-sensitivity C-reactive protein (hsCRP) levels. In addition, we constructed an additive interaction model to study whether there was an additive interaction between sedentary behavior and depressive symptoms in relation to MAFLD risk. The rationale for investigating this interaction is that existing relevant studies have shown the association between sedentary behavior, depressive symptoms, and metabolic syndrome, confirming that depression plays a mediating role between sedentary behavior and metabolic disorders [20]. We prioritized additive interaction analysis because it better reflects biological synergy and aligns with our core goal of assessing whether the combined effect of the two factors exceeds the sum of their individual effects on MAFLD risk. The additive interaction between sedentary behavior and depression related to MAFLD was measured by estimating whether the combined effect of the two factors was greater than the sum of their independent effects. The relative excess risk due to interaction (RERI), attributable proportion due to interaction (AP), and synergy index (S) are used to assess additive interactions. The analysis of these three additive interaction indicators covers the unadjusted model (Model 1), partially adjusted model (Model 2), and fully adjusted model (Model 3) mentioned earlier, to comprehensively explore the additive interaction effects among variables under different levels of confounding factor adjustment. When the 95% confidence intervals (CIs) for RERIs and APs include 0 and the 95% confidence interval for S includes 1, there is no additive interaction. Subgroup analyses were further conducted on the basis of body mass index. All the statistical tests were two-sided and performed via R v.4.3.2 software. We incorporated NHANES’ complex sampling characteristics (sampling weights, primary sampling units, and strata) into all analyses, including descriptive statistics (weighted prevalence/means), group comparisons (weighted chi-square test, weighted t-test), multivariate regression models, additive interaction analyses, and subgroup analyses. A *P* value of < 0.05 was considered statistically significant.

### Ethical statement

The Ethics Review Board of the National Center for Health Statistics granted ethical approval for this research. All procedures were conducted in compliance with pertinent guidelines and regulations (Declaration of Helsinki). Written informed consent was obtained from all individuals prior to their participation in the study.

## Results

**Statistical description of the study population:** A total of 10,612 research subjects were included according to the inclusion and exclusion criteria. The participants were divided into non-MAFLD (n = 6111, 58% weighted prevalence) and the MAFLD group (n = 4501, 42% weighted prevalence) on the basis of the MAFLD diagnostic criteria. For our variables of interest, the MAFLD group had a longer average sedentary time (377.15 vs. 347.43 min/d) and higher PHQ-9 scores (3.55 vs. 3.14) compared with the non-MAFLD group, with statistically significant differences (both P value < 0.05). Differences in other demographic, lifestyle and clinical covariates (e.g., sex, age, BMI, diabetes) are presented in Table 1. The overall characteristics of the participants classified by MAFLD are shown in Table 1. The missing data in this study mainly focused on resting behavior, depression (PHQ-9 score), liver elasticity and some covariates (such as educational level, smoking status, BMI, etc.). Among them, liver elasticity was the target detection index and was only detected in some participants, which was a selective variable missing. The absence of resting behavior, PHQ-9 score and physiological indicators (such as HDL, TC) mostly results from non-filling of the questionnaire or non-cooperation in the test, which is a random absence. The absence of demographic characteristics (such as educational level) is extremely rare and sporadic. Through weighted linear regression (for continuous variables) and weighted chi-square test (for categorical variables) analysis, there were statistically significant differences between the two groups in most features (P < 0.001), and the core differences are as follows (S1 Table): The included group was older (48.21 ± 16.99 vs 45.97 ± 20.23), had a higher proportion of non-Hispanic whites (63.8% vs 54.4%), and had a higher proportion of those with an educational level above high school (64.8% vs 55.4%). The proportion of previous smokers in the included group was higher (15.6% vs 10.6%), while the proportion of current smokers was slightly lower (25.1% vs 26.5%). The proportion of overweight was higher in the included group (32.7% vs 27.6%), the prevalence of MAFLD was higher (41.8% vs 38.8%, P = 0.008), and the prevalence of diabetes was lower (13.8% vs 16.5%) The level of hsCRP was lower (3.63 ± 7.13 vs 4.35 ± 8.70); There was no difference in sedentary time and classification distribution between the two groups, but the PHQ-9 score was higher in the exclusion group (4.28 ± 4.83 vs 3.31 ± 4.15), and the risk of depression was higher.

**Table 1:** Group differences between the MAFLD and non-MAFLD groups.

Characteristic	Overall N = 10612^1^	Non-MAFLD N = 6111 (58%)^1^	MAFLD N = 4501 (42%)^1^	*P* value^2^
Age, n (%)^1^	48.21 (16.99)	46.22 (17.47)	50.97 (15.88)	<0.001
Sex, n (%)^1^				<0.001
Female	5497 (50%)	3402 (55%)	2095 (44%)	
Male	5115 (50%)	2709 (45%)	2406 (56%)	
Race, n (%)^1^				<0.001
Mexican American	1059 (7.9%)	456 (6.1%)	603 (10%)	
Other Hispanic	1096 (8.2%)	618 (8.3%)	478 (8.0%)	
Non-Hispanic White	4891 (64%)	2803 (64%)	2088 (64%)	
Non-Hispanic Black	2047 (10%)	1326 (11%)	721 (8.3%)	
Non-Hispanic Asian	940 (5.4%)	570 (5.7%)	370 (5.0%)	
Other/multiracial	579 (4.7%)	338 (4.7%)	241 (4.8%)	
Education level, n (%)^1^				<0.001
High school or below	3881 (35%)	2104 (33%)	1777 (39%)	
High school above	6726 (65%)	4005 (67%)	2721 (61%)	
Others	5 (<0.1%)	2 (<0.1%)	3 (<0.1%)	
Smoke, n (%)^1^				<0.001
Never	6230 (59%)	3703 (62%)	2527 (56%)	
Former	1433 (16%)	689 (13%)	744 (19%)	
Current	2949 (25%)	1719 (25%)	1230 (25%)	
BMI, n (%)^1^				<0.001
Normal or lighter	2824 (27%)	2504 (43%)	320 (6.4%)	
Overweight	3480 (33%)	2189 (36%)	1291 (28%)	
Obesity	4308 (40%)	1418 (21%)	2890 (66%)	
Sedentary Time (min/d)	359.85 (206.42)	347.43 (201.83)	377.15 (211.47)	<0.001
Sedentary Time Category, n (%)^1^				<0.001
< 2 hours	1760 (14%)	1111 (17%)	649 (12%)	
2 hours ~ 6 hours	5244 (49%)	3021 (49%)	2223 (49%)	
> 6 hours	3608 (37%)	1979 (34%)	1629 (39%)	
Diabetes, n (%)^1^				<0.001
No	8699 (86%)	5487 (93%)	3212 (76%)	
Yes	1913 (14%)	624 (6.6%)	1289 (24%)	
HDL (mmol/L)	1.39 (0.40)	1.49 (0.40)	1.26 (0.35)	<0.001
TC (mmol/L)	4.87 (1.06)	4.82 (1.03)	4.96 (1.09)	<0.001
hsCRP (mg/L)	3.63 (7.13)	2.89 (6.98)	4.67 (7.21)	<0.001
PHQ-9 score (n)	3.31 (4.15)	3.14 (4.03)	3.55 (4.31)	<0.001
PHQ-9 score category, n (%)^1^				0.005
< 5	7713 (74%)	4529 (75%)	3184 (71%)	
5-9	1869 (17%)	1032 (16%)	837 (19%)	
> 9	1030 (9.1%)	550 (8.5%)	480 (9.8%)	

1 Mean (SD); n (unweighted) (%).

2 The *P* values for continuous variables were derived using a weighted linear regression analysis, while categorical variables were analyzed through a weighted chi-square test.

**Fig 2:**
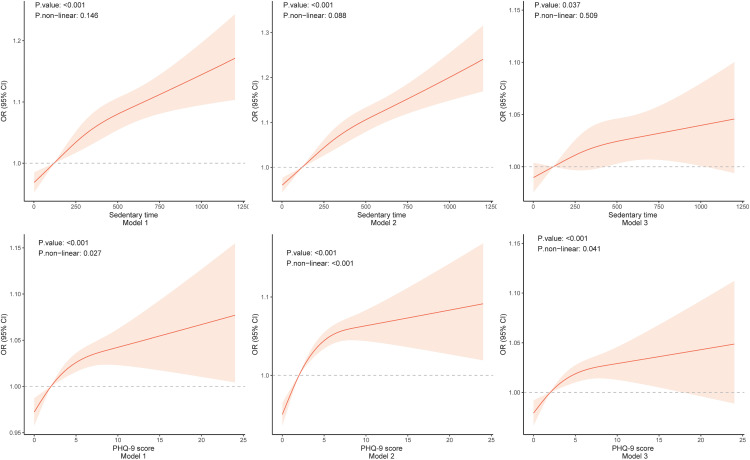
Associations between sedentary time, PHQ-9 score and odds ratio (OR) of MAFLD under different models, with the red shading in the Figs indicating the 95% confidence interval for the OR.

**Correlation between sedentary behavior and MAFLD:** Compared with short sedentary times (<2 hours/day, reference group), moderate (2–6 hours/day) and long (>6 hours/day) sedentary times exhibited a significantly higher risk of MAFLD. This trend was statistically significant in both the minimally adjusted model and the fully adjusted model (Table 2). The fully adjusted model results revealed that the OR for moderate sedentary time was 1.26 (95% CI: 1.03 ~ 1.55) and that for long sedentary time was 1.33 (95% CI: 1.08 ~ 1.64, *P* value 0.011). For continuous sedentary time, we further used restricted cubic spline plots to explore its association with MAFLD risk (Fig 2). Additionally, results from the fully adjusted model using multiple imputation data showed consistent trends: the OR for moderate sedentary time was 1.26 (95% CI: 1.03 ~ 1.55, P = 0.025) and for long sedentary time was 1.33 (95% CI: 1.08 ~ 1.64, P = 0.007). These findings were highly consistent with those from complete case analysis (CCA), confirming the robustness of the conclusion (S2 Table).

**Table 2:** Associations between sedentary time category and MAFLD, NHANES 2017–2020, 2021-2023.

Characteristic	Model 1 OR (95% CI)	Model 2 aOR (95% CI)	Model 3 aOR (95% CI)
Sedentary Time Category			
< 2 hours	Reference	Reference	Reference
2 hours ~ 6 hours	1.43(1.21, 1.69)	1.49(1.26, 1.76)	1.26(1.03, 1.55)
> 6 hours	1.64(1.36, 1.97)	1.90(1.59, 2.28)	1.33(1.08, 1.64)
*P* value	<0.001	<0.001	0.011

Model 1: no covariates were adjusted. Model 2: Age, sex, race and education level were adjusted for. Model 3 was adjusted for age, sex, race, education level, BMI, smoking status, HDL-C, total cholesterol, hsCRP and diabetes.

**Correlation between depression and MAFLD:** Compared with a PHQ-9 score < 5, a PHQ-9 score ≥ 5 was positively correlated with the risk of MAFLD. This correlation was statistically significant in both the minimally adjusted model and the fully adjusted model (Table 3). The fully adjusted model results revealed that the OR for PHQ-9 scores between 5 and 9 was 1.24 (95% CI: 1.01 ~ 1.53) and that for scores > 9 was 1.26 (95% CI: 1.03 ~ 1.55, *P* value 0.006). Restricted cubic spline plots revealed a nonlinear correlation between PHQ-9 scores and the risk of MAFLD (Fig 2). Meanwhile, results from the fully adjusted model using multiple imputation data showed that mild depression was associated with a non-statistically significant increase in MAFLD risk (OR = 1.20, 95% CI: 0.99 ~ 1.46, P = 0.067), whereas moderate-severe depression remained significantly associated with elevated MAFLD risk (OR = 1.24, 95% CI: 1.02 ~ 1.50, P = 0.035). Despite the lack of statistical significance for mild depression in imputed data, the overall trend of depression-related MAFLD risk elevation was consistent with the findings from complete case analysis (CCA), further verifying the robustness of the core conclusion (S2 Table).

**Table 3:** Associations between PHQ-9 score categories and MAFLD, NHANES 2017–2020, 2021-2023.

Characteristic	Model 1 OR (95% CI)	Model 2 aOR (95% CI)	Model 3 aOR (95% CI)
PHQ-9 Score Category			
< 5	Reference	Reference	Reference
5-9	1.39(1.16, 1.67)	1.26(1.06, 1.50)	1.24(1.01, 1.53)
> 9	1.42(1.17, 1.71)	1.22(1.03, 1.46)	1.26(1.03, 1.55)
*P* value	0.006	<0.001	0.011

Model 1: no covariates were adjusted. Model 2: Age, sex, race and education level were adjusted for. Model 3 was adjusted for age, sex, race, education level, BMI, smoking status, HDL-C, total cholesterol, hsCRP and diabetes.

**Interaction effect of sedentary behavior and depression on MAFLD:** According to the PHQ-9 scoring criteria, a score exceeding 5 points indicates mild depression, whereas a score surpassing 9 points suggests moderate-to-severe depression [15]. On the basis of our findings (Fig 2), we observed a nonlinear correlation between PHQ-9 scores and the risk of MAFLD. Therefore, individuals with PHQ-9 scores equal to or greater than 5 were classified as exhibiting depressive symptoms. As shown in Table 4, in Model 3, there was no additive interaction effect between long sedentary behavior and depression on MAFLD (adjusted RERI for long sedentary behavior and depression on MAFLD = −0.063, 95% CI = −0.496 ~ 0.370; adjusted AP = −0.037, 95% CI = −0.093 ~ 0.019; adjusted S = 0.891, 95% CI = 0.700 ~ 1.082).

**Table 4:** Interactive effect analysis of sedentary behavior and depression, NHANES 2017–2020, 2021-2023.

Characteristic		Model 1 OR (95% CI)	Model 2 aOR (95% CI)	Model 3 aOR (95% CI)
Sedentary	Depression			
< 2 hours	No	Reference	Reference	Reference
2 hours ~ 6 hours	No	1.44(1.16, 1.77)	1.50(1.19, 1.88)	1.31(1.02, 1.70)
> 6 hours	No	1.56(1.21, 2.01)	1.82(1.40, 2.35)	1.36(1.01, 1.84)
< 2 hours	Yes	1.17(0.85, 1.59)	1.33(0.94, 1.89)	1.40(0.97, 2.02)
2 hours ~ 6 hours	Yes	1.68(1.30, 2.17)	1.94(1.49, 2.53)	1.57(1.13, 2.19)
> 6 hours	Yes	2.16(1.72, 2.71)	2.77(2.22, 3.45)	1.70(1.31, 2.20)
*P* value		<0.001	<0.001	<0.001
RERI		0.435(−0.013,0.884)	0.622(0.090,1.153)	−0.063(−0.496,0.370)
AP		0.201(0.154,0.249)	0.224(0.172,0.276)	0.037(−0.093,0.019)
S		1.189(0.930,1.448)	1.145(0.930,1.360)	0.891(0.700,1.082)

Model 1: no covariates were adjusted. Model 2: Age, sex, race and education level were adjusted for. Model 3 was adjusted for age, sex, race, education level, BMI, smoking status, HDL-C, total cholesterol, hsCRP and diabetes.

**Interaction between sedentary behavior and depression on MAFLD in different BMI groups:** Considering the strong correlation between MAFLD and weight, we divided participants into normal or lighter weight groups (BMI < 25 kg/m^2^), overweight groups (25 kg/m^2^ ≤ BMI < 30 kg/m^2^), and obese groups (BMI > 30 kg/m^2^). As shown in Table 5, in overweight MAFLD patients in Model 3, there was a significant synergistic effect between long sedentary behavior and depression on MAFLD (adjusted RERI for long sedentary behavior and depression on MAFLD = 0.684, 95% CI = 0.312 ~ 1.057; adjusted AP = 0.360, 95% CI = 0.323 ~ 0.397; adjusted S = 1.623, 95% CI = 1.177 ~ 2.070). Among them, the value of AP in Model 3 was 0.360, indicating that 36.0% of the overweight MAFLD cases in this study sample were caused by the interaction effect of sedentary behavior and depression. However, considering the high prevalence of MAFLD in the study sample, the interaction effect estimated by logistic regression might be overestimated. To address this potential bias, we re-calculated the interaction effect using Poisson regression. When a 95% CI was applied in Poisson regression analyses, no significant interaction effects were observed across all BMI subgroups (S3 Table). Further exploration with a 90% CI revealed a potential interaction effect in the overweight subgroup (RERI = 0.318, 90% CI: 0.036 ~ 0.600; AP = 0.223, 90% CI: 0.182 ~ 0.263; S = 1.310, 90% CI: 1.000 ~ 1.620), suggesting that sedentary behavior may still interact with depression to influence MAFLD risk in overweight individuals (S4 Table).

**Table 5:** Interactive effect analysis of sedentary behavior and depression in participants stratified by body mass index.

Characteristic		BMI		
		Normal or lower (27.4%)	Overweight (32.7%)	Obesity (39.9%)
Sedentary	Depression			
< 2 hours	No	Reference	Reference	Reference
2 hours ~ 6 hours	No	1.31(0.80, 2.15)	1.16(0.77, 1.77)	1.47(1.06, 2.02)
> 6 hours	No	1.11(0.65, 1.91)	1.34(0.82, 2.19)	1.57(1.09, 2.25)
< 2 hours	Yes	1.12(0.55, 2.32)	0.87(0.54, 1.41)	2.49(1.50, 4.14)
2 hours ~ 6 hours	Yes	1.14(0.46, 2.82)	1.41(0.92, 2.19)	1.97(1.30, 2.99)
> 6 hours	Yes	0.67(0.23, 1.94)	1.90(1.19, 3.04)	2.00(1.35, 2.97)
*P* value		0.6	0.002	0.001
RERI		−0.564(−0.886, −0.245)	0.684(0.312, 1.057)	−1.054(−1.680, −0.427)
AP		−0.837(−0.915, −0.759)	0.360(0.323, 0.397)	−0.526(−0.626, −0.426)
S		0.538(0.378, 0.698)	1.623(1.177, 2.070)	0.513(0.432, 0.595)

The results were obtained via fully adjusted Model 3. RERI indicates the relative excess risk due to interaction. AP stands for the attributable proportion of interaction. S denotes the synergy index. OR represents the odds ratio. CI signifies the confidence interval.

## Discussion

In this study, we used data from 10,612 people in NHANES 2017 ~ 2020 and 2021 ~ 2023 to study the relationships between depression and sedentary behavior and their interactive effects on MAFLD. We found that sedentary behavior and depression are independently associated with increased MAFLD risk, and depression and sedentary behavior might have an additive interactive effect on the risk of MAFLD in overweight individuals.

First, this study revealed a significant positive correlation between sedentary time and depression with MAFLD in the entire study population after adjusting for age, sex, race, education level, BMI, smoking status, diabetes status, and plasma high-density lipoprotein cholesterol (HDL-C), total cholesterol (TC), and high-sensitivity C-reactive protein (hsCRP) levels. Moreover, this study identified a potential additive interactive effect of depression and sedentary behavior on MAFLD risk specifically in overweight individuals following the same covariate adjustment. To address concerns about the potential discrepancy between odds ratios (ORs) and risk ratios (RRs) due to the relatively high MAFLD prevalence (42%), we supplemented Poisson regression with robust variance estimation for verification. While the interaction effect did not reach statistical significance with a 95% confidence interval (CI) across all BMI subgroups (consistent with the conservative nature of 95% CI for common outcomes), the 90% CI confirmed a meaningful additive interaction in overweight participants (RERI = 0.318, 90% CI: 0.036 ~ 0.600; AP = 0.223, 90% CI: 0.182 ~ 0.263; S=1.310, 90% CI: 1.000 ~ 1.620). This finding aligns with the trend observed in logistic regression analyses. Additionally, considering that the MAFLD prevalence in the included population was slightly higher than in the excluded group, the estimated interaction effect might be moderately attenuated compared to the true effect. Collectively, these consistent trends across analytical approaches support the presence of a synergistic effect of depression and sedentary behavior on MAFLD risk in overweight individuals.

Previous studies have revealed a correlation between depression and MAFLD in NHANES data. In addition, scholars have reported a positive correlation between depression and NAFLD in German and Korean populations [21,22]. This finding is similar to the results of the present study. However, unlike the results of previous studies, our study revealed a nonlinear relationship between PHQ-9 scores and the risk of developing MAFLD. Specifically, when a PHQ-9 score > 5 indicates mild depression, the risk of MAFLD increases accordingly. Therefore, the standard for determining the presence of depressive symptoms in subsequent calculations of the interactive effects of depression and sedentary behavior on MAFLD is a PHQ score ≥5. Sedentary behavior is an independent risk factor for many metabolic diseases. Previous studies have shown a dose‒response relationship between sedentary time and NAFLD (*P* for trend < 0.001) [23]. Moreover, recent studies have shown that sedentary behavior is associated with the risk of MAFLD-related cardiovascular disease [24]. This study, the first to include NHANES data from 2021 ~ 2023, provides an analysis of the association between sedentary behavior and MAFLD and reveals that people who are sedentary for a long time (more than 6 hours per day) have a greater risk for MAFLD (OR: 1.33, 95% confidence interval: 1.08--1.64).

Depressive states may affect an individual’s metabolic health by activating inflammatory responses and disrupting endocrine balance, which may lead to insulin resistance and lipid metabolism abnormalities, thereby increasing the risk of MAFLD [25–27]. In addition, studies have confirmed a close link between sedentary behavior and metabolic abnormalities [28]. Specifically, long periods of sedentary behavior may lead to a significant increase in postprandial blood glucose, insulin, and triglyceride levels, which may be due to reduced muscle activity from sitting, promoting abnormal fat accumulation, and ultimately leading to insulin resistance and dyslipidemia, all of which are risk factors for MAFLD [29,30].

Bidirectional relationships may exist: depression reduces motivation for physical activity, increasing sedentary behavior [11]; prolonged sedentary behavior disrupts metabolic and inflammatory pathways, exacerbating depression [25]. Similarly, MAFLD may induce depression via systemic inflammation [6], while depression worsens MAFLD via metabolic dysregulation [26]. However, this cross-sectional study cannot confirm causality—prospective studies are needed to clarify directionality.

Considering that depressive states and sedentary behavior may both be risk factors for MAFLD, sedentary behavior may be a risk factor for depressive states. When sedentary behavior is present, it may lead to the simultaneous occurrence of depressive states and MAFLD. In our study, we found that the coexistence of long sedentary behavior and depression had a significant additive effect on the risk of MAFLD in overweight individuals. These findings suggest that reducing sedentary time and maintaining a pleasant mood should be helpful for overweight MAFLD patients. Moreover, we did not find a similar additive interaction effect in MAFLD patients with normal or lighter weight or obesity, possibly because MAFLD in these participants is more common for other reasons.

**Strengths and Limitations:** Strengths: Included latest NHANES 2021 ~ 2023 data; incorporated complex sampling design and key confounders; subgroup analysis by BMI provided targeted insights. Limitations: Cross-sectional design cannot infer causality; potential residual confounding; self-reported sedentary behavior may introduce recall bias; MAFLD diagnosed via non-invasive CAP instead of histopathology limits severity assessment.

For clinicians, incorporating brief mental health screening (such as PHQ-9) into the management of Metabolically Associated Fatty Liver Disease (MAFLD) and providing targeted advice on reducing sedentary time (such as using sit-stand workstations or regular activity breaks) may be a synergistic strategy to reduce the risk of liver disease.

## Conclusion

Our study revealed a synergistic effect of sedentary behavior and depressive symptoms on metabolic-associated liver disease (MAFLD) in overweight individuals. This means that when these two factors coexist, their promoting effects on MAFLD may be amplified. Our results emphasize the importance of identifying and intervening in sedentary behavior and depressive symptoms in overweight populations. However, more prospective studies are needed to confirm our current results.

## Supporting information

S1 Table Baseline Characteristics of the Study Population (Overall, Exclusion Group vs. Included Group). (DOCX)

S2 Table Associations Between Sedentary Behavior, Depression, and MAFLD in Model 3 (After Multiple Imputation, Fully Adjusted). (DOCX)

S3 Table Interactive effect analysis of sedentary behavior and depression on MAFLD risk(with 95% confidence intervals), stratified by body mass index (Poisson regression). (DOCX)

S4 Table Interactive effect analysis of sedentary behavior and depression on MAFLD risk in the Overweight Group (with 90% confidence intervals), stratified by body mass index (Poisson regression). (DOCX)

## Abbreviations

NHANES: National Health and Nutritional Examination Survey
SD: standard deviation
CI: confidence interval
MAFLD: metabolic-associated fatty liver disease
NAFLD: nonalcoholic fatty liver disease
BMI: body mass index
hsCRP: high-sensitivity C-reactive protein
HDL-C: high-density lipoprotein cholesterol
TC: total cholesterol
PHQ-9: Patient Health Questionnaire
RERI: relative excess risk due to interaction
AP: attributable proportion of interaction
S: synergy index
