# Supplementary material for: Interaction effects of sedentary behavior and depression on MAFLD in NHANES 2017–2020 and 2021–2023
Source: PLoS One. 2026 Feb 17;21(2):e0342336. doi: 10.1371/journal.pone.0342336 (PMC12912620; doi:10.1371/journal.pone.0342336)
Supplement: S1 Table — (DOCX) [file pone.0342336.s001.docx]

**S1 Table:**

Baseline Characteristics of the Study Population (Overall, Exclusion Group vs. Included Group).

| **Characteristic** | Overall  (n = 250136202)^1^ | Exclusion group  (n = 66904609)^1^ | Included group  (n = 183231592)^1^ | ***P* value**^2^ |
| --- | --- | --- | --- | --- |
| Age, n (%)^1^ | 47.61 (17.94) | 45.97 (20.23) | 48.21 (16.99) | <0.001 |
| Sex, n (%)^1^ |  |  |  | <0.001 |
| Female | 129,189,684 (51.6%) | 37,258,917 (55.7%) | 91,930,767 (50.2%) |  |
| Male | 120,946,518 (48.4%) | 29,645,692 (44.3%) | 91,300,826 (49.8%) |  |
| Race, n (%)^1^ |  |  |  | <0.001 |
| Mexican American | 20,181,592 (8.1%) | 5,784,107 (8.6%) | 14,397,485 (7.9%) |  |
| Other Hispanic | 21,197,182 (8.5%) | 6,214,768 (9.3%) | 14,982,414 (8.2%) |  |
| Non-Hispanic White | 153,254,181 (61.3%) | 36,401,637 (54.4%) | 116,852,544 (63.8%) |  |
| Non-Hispanic Black | 28,185,539 (11.3%) | 9,713,634 (14.5%) | 18,471,904 (10.1%) |  |
| Non-Hispanic Asian | 15,067,810 (6.0%) | 5,148,556 (7.7%) | 9,919,254 (5.4%) |  |
| Other/multiracial | 12,249,899 (4.9%) | 3,641,907 (5.4%) | 8,607,993 (4.7%) |  |
| Education level, n (%)^1^ |  |  |  | <0.001 |
| High school or below | 90,682,657 (37.4%) | 26,182,578 (44.4%) | 64,500,078 (35.2%) |  |
| High school above | 151,366,836 (62.5%) | 32,680,314 (55.4%) | 118,686,522 (64.8%) |  |
| Others | 173,722 (0.1%) | 128,729 (0.2%) | 44,992 (0.0%) |  |
| Missing | 7,912,988 | 7,912,988 | 0 |  |
| Smoke, n (%)^1^ |  |  |  | <0.001 |
| Never | 150,831,514 (60.3%) | 42,134,750 (63.0%) | 108,696,764 (59.3%) |  |
| Former | 35,670,726 (14.3%) | 7,057,437 (10.6%) | 28,613,289 (15.6%) |  |
| Current | 63,623,808 (25.4%) | 17,702,268 (26.5%) | 45,921,540 (25.1%) |  |
| Missing | 10,154 | 10,154 | 0 |  |
| MAFLD | 96,496,201 (41.1%) | 19,905,112 (38.8%) | 76,591,089 (41.8%) | 0.008 |
| Missing | 15,625,830 | 15,625,830 | 0 |  |
| BMI, n (%)^1^ |  |  |  | <0.001 |
| Normal or lighter | 70,034,190 (28.4%) | 19,725,772 (31.0%) | 50,308,418 (27.5%) |  |
| Overweight | 77,432,114 (31.4%) | 17,557,506 (27.6%) | 59,874,607 (32.7%) |  |
| Obesity | 99,350,831 (40.3%) | 26,302,263 (41.4%) | 73,048,567 (39.9%) |  |
| Missing | 3,319,068 | 3,319,068 | 0 |  |
| Sedentary Time (min/day) | 359.06 (206.36) | 356.86 (206.17) | 359.85 (206.42) | 0.630 |
| Missing | 1,576,489 | 1,576,489 | 0 |  |
| Sedentary Time Category, n (%)^1^ |  |  |  | 0.559 |
| < 2 hours | 36,017,885 (14.5%) | 9,461,076 (14.5%) | 26,556,810 (14.5%) |  |
| 2 hours ~ 6 hours | 122,563,657 (49.3%) | 32,827,435 (50.3%) | 89,736,222 (49.0%) |  |
| >6 hours | 89,978,171 (36.2%) | 23,039,610 (35.3%) | 66,938,561 (36.5%) |  |
| Missing | 1,576,489 | 1,576,489 | 0 |  |
| Diabetes, n (%)^1^ |  |  |  | <0.001 |
| No | 213,835,233 (85.5%) | 55,844,297 (83.5%) | 157,990,935 (86.2%) |  |
| Yes | 36,300,970 (14.5%) | 11,060,312 (16.5%) | 25,240,658 (13.8%) |  |
| HDL (mmol/L) | 1.39 (0.39) | 1.37 (0.38) | 1.39 (0.40) | 0.093 |
| Missing | 18,266,506 | 18,266,506 | 0 |  |
| TC (mmol/L) | 4.83 (1.07) | 4.68 (1.11) | 4.87 (1.06) | <0.001 |
| Missing | 18,266,506 | 18,266,506 | 0 |  |
| hsCRP (mg/L) | 3.79 (7.49) | 4.35 (8.70) | 3.63 (7.13) | <0.001 |
| Missing | 17,709,420 | 17,709,420 | 0 |  |
| PHQ-9 score(n) | 3.50 (4.31) | 4.28 (4.83) | 3.31 (4.15) | <0.001 |
| Missing | 22,083,446 | 22,083,446 | 0 |  |
| PHQ-9 score category, n (%)^1^ |  |  |  | <0.001 |
| <5 | 164,389,643 (72.1%) | 29,521,665 (65.9%) | 134,867,978 (73.6%) |  |
| 5-9 | 40,731,194 (17.9%) | 9,000,463 (20.1%) | 31,730,731 (17.3%) |  |
| >9 | 22,931,919 (10.1%) | 6,299,035 (14.1%) | 16,632,884 (9.1%) |  |
| PHQ-9 score(n) | 22,083,446 | 22,083,446 | 0 |  |
| ^1^Mean (SD); n (%) | | | | |
| ^2^ The p values for continuous variables were derived using a weighted linear regression analysis, while categorical variables were analyzed through a weighted chi-square test. | | | | |
