# Supplementary material for: Interaction effects of sedentary behavior and depression on MAFLD in NHANES 2017–2020 and 2021–2023
Source: PLoS One. 2026 Feb 17;21(2):e0342336. doi: 10.1371/journal.pone.0342336 (PMC12912620; doi:10.1371/journal.pone.0342336)
Supplement: S2 Table — (DOCX) [file pone.0342336.s002.docx]

**S2 Table:**

Associations Between Sedentary Behavior, Depression, and MAFLD in Model 3 (After Multiple Imputation, Fully Adjusted).

| **Characteristic** | OR(95%CI) | *P* value |
| --- | --- | --- |
| Sedentary Time Category | - | - |
| < 2 hours (Ref) | 1.00 (Ref) | - |
| 2 hours ~ 6 hours | 1.26 (1.03, 1.55) | 0.025 |
| > 6 hours | 1.33 (1.08, 1.64) | 0.007 |
| PHQ-9 Category | - | - |
| No depression (Reference) | 1.00 (Reference) | - |
| Mild depression | 1.20 (0.99, 1.46) | 0.067 |
| Moderate-severe depression | 1.24 (1.02, 1.50) | 0.035 |

Model 3: age, gender, race, education level, BMI, smoking status, HDL-cholesterol, Total-cholesterol, hsCRP and diabetes were adjusted.
