# Supplementary material for: Interaction effects of sedentary behavior and depression on MAFLD in NHANES 2017–2020 and 2021–2023
Source: PLoS One. 2026 Feb 17;21(2):e0342336. doi: 10.1371/journal.pone.0342336 (PMC12912620; doi:10.1371/journal.pone.0342336)
Supplement: S3 Table — (DOCX) [file pone.0342336.s003.docx]

**S3 Table**：

| **Characteristic** | | BMI | | |
| --- | --- | --- | --- | --- |
|  |  | Normal or lower (27.4%) | Overweight  (32.7%) | Obesity  (39.9%) |
| Sedentary | Depression |  |  |  |
| < 2 hours | No | Reference | Reference | Reference |
| 2 hours ~  6 hours | No | 1.25  (0.84, 1.85) | 1.10  (0.85, 1.41) | 1.14  (1.02, 1.27) |
| >6 hours | No | 1.12  (0.72, 1.74) | 1.20  (0.90, 1.60) | 1.16  (1.02, 1.31) |
| < 2 hours | Yes | 1.14  (0.62, 2.11) | 0.91  (0.67, 1.23) | 1.30  (1.12, 1.51) |
| 2 hours ~  6 hours | Yes | 1.12  (0.53, 2.37) | 1.23  (0.95, 1.59) | 1.24  (1.08, 1.41) |
| >6 hours | Yes | 0.71  (0.28, 1.78) | 1.43  (1.09, 1.86) | 1.24  (1.10, 1.40) |
| *P* value |  | 0.6 | 0.002 | <0.001 |
| RERI |  | -0.551(-0.878,-0.224) | 0.318(-0.018, 0.655) | -0.214(-0.589, 0.161) |
| AP |  | -0.837(-0.851,-0.698) | 0.223(0.182, 0.263) | -0.172(-0.230, -0.115) |
| S |  | 0.538(0.393, 0.718) | 1.310(0.936, 1.681) | 0.826(0.621, 1.030) |

Interactive effect analysis of sedentary behavior and depression on MAFLD risk(with 95% confidence intervals), stratified by body mass index (Poisson regression).

The results were obtained using the fully adjusted Model 3. RERI indicates the relative excess risk due to interaction. AP stands for the attributable proportion of interaction. S denotes the synergy index. OR represents the odds ratio. CI signifies the confidence interval.
