# Supplementary material for: Interaction effects of sedentary behavior and depression on MAFLD in NHANES 2017–2020 and 2021–2023
Source: PLoS One. 2026 Feb 17;21(2):e0342336. doi: 10.1371/journal.pone.0342336 (PMC12912620; doi:10.1371/journal.pone.0342336)
Supplement: S4 Table — (DOCX) [file pone.0342336.s004.docx]

**S4 Table：**

Interactive effect analysis of sedentary behavior and depression on MAFLD risk in the Overweight Group (with 90% confidence intervals), stratified by body mass index (Poisson regression).

| **Characteristic** | | **Overweight Group** |
| --- | --- | --- |
| Sedentary | Depression |  |
| < 2 hours | No | Reference |
| 2 hours ~6 hours | No | 1.10  (0.89, 1.35) |
| >6 hours | No | 1.20  (0.94, 1.52) |
| < 2 hours | Yes | 0.91  (0.71, 1.17) |
| 2 hours ~ 6 hours | Yes | 1.23  (0.99, 1.52) |
| >6 hours | Yes | 1.43  (1.14, 1.78) |
| *P* value |  | 0.002 |
| RERI |  | 0.318(0.036, 0.600) |
| AP |  | 0.223(0.189, 0.257) |
| S |  | 1.310(1.000, 1.620) |

The results were obtained using the fully adjusted Model 3. RERI indicates the relative excess risk due to interaction. AP stands for the attributable proportion of interaction. S denotes the synergy index. OR represents the odds ratio. CI signifies the confidence interval.
